# Father’s Knowledge, Attitude and Support to Mother’s Exclusive Breastfeeding Practices in Bangladesh: A Multi-Group Structural Equations Model Analysis

**DOI:** 10.3390/healthcare9030276

**Published:** 2021-03-03

**Authors:** Yan-Qiong Ouyang, Layla Nasrin

**Affiliations:** School of Health Sciences, Wuhan University, Wuhan 430072, China; ouyangyq@whu.edu.cn

**Keywords:** knowledge, attitude, support, exclusive breastfeeding, theory of planned behavior, structural equation model

## Abstract

Despite worldwide initiatives, the exclusive breastfeeding (EBF) rate is low. The study aims to investigate the role of fathers’ knowledge, attitude and support in formulating mothers’ practice of breastfeeding taking Bangladeshi parental cases as a sample. The study uses a standard survey instrument following the theory of planned behavior (TPB). Responses from 332 couples are accepted following a standard criteria and used for analysis. The sample is divided into sub-groups based on delivery mode—vaginal (*n* = 211, 64%) and cesarean section (*n* = 121, 36%). Based on the sub-groups, a multi-group structural equation modelling (SEM) is applied to analyze the phenomena. The study finds that a father’s knowledge in EBF can, in one way, significantly enhance mother’s knowledge by sharing and, in another way, can enhance his own attitude to offer different support to his partner/wife which induces the chances of EBF practices by mothers. The enhanced EBF knowledge of a mother raises her attitude to practice EBF, which is also positively affected by the father’s attitude. Thus, fathers’ support, coupled with mothers’ positive attitude, the mother’s intention to practice EBF as per standard guidelines.

## 1. Introduction

All over the world, breastfeeding is recognized as the prime nutrition gaining source for the newborn [1,2], at least for the first six months postpartum. The World Health Organization (WHO) also recommended the first six months of birth for exclusive breastfeeding (EBF), considering the key role of breastfeeding for child development and growth, as well as the health benefits of EBF for mothers. 

Exclusive breastfeeding can protect the baby from allergies, obesity, constipation, diarrhea and also reduce the risk of childhood infections [3]. Moreover, breastfeeding can help to improve cognitive and motor development [4] and decrease sudden infant death syndrome (SIDS) rates [5]. On the other hand, practicing EBF can protect the mothers from type II diabetes and more importantly ovarian and breast cancers [6,7]. Previous literature reports that exclusively breastfed children are at lower risk of having acute respiratory and gastrointestinal infections compared with children who are not breastfed exclusively [8]. In high human immunodeficiency virus (HIV) prevalence settings, EBF is found to prevent 13% of child deaths [9]. It has also been shown that the rate of HIV transmission from mother to child is lower in exclusively breastfed children compared with non-EBF children [10].

Despite the inevitable benefits of EBF, at under 1 month, the global rate of EBF is only 50%, while the rate for infants aged between 1 to 5 months is only 30% [11]. According to the global breastfeeding scorecard 2019, an overall global prevalence of EBF up to six months is 41%, whereas the initiation of breastfeeding at first hour of birth is only 43% [12]. The determining factors for practicing EBF is found to vary between countries and even varies within the same country.

The rate of EBF practice, in Bangladesh, a lower-middle-income country [13], was found to be 55% in 2014 [1,14]. Although the rate in 2014 was above the world average, it has fluctuated a lot historically. For example, in 1993 to 2000, the rate was around 42%, in Bangladesh Demographic and Health Surveys in 2004, 2007, 2011 and 2014 the rate was 42%, 43%, 64%, and 55%, respectively. A further decline was noticed with only 47% in 2015, according to ‘The National Food Policy Plan of Action and Country Investment Plan’ of the food ministry released in 2017 [15]. Such a decline in the EBF rate was our main motivation to investigate the knowledge and attitude of fathers and mothers of this country which may have a significant influence on building the intention of mothers to continue EBF up to six months.

The decline in the EBF rate in Bangladesh may have happened due to discontinuance of activities to promote EBF practices which took place in around 2011 [16]. Lack of paternal and family support, workplace barriers [2,17] such as shorter maternity leave [17], breastfeeding in unfriendly work environments (as most offices do not have daycare centers and do not allow time and/or space for expressing breastmilk) and long working hours [16] etc. also may lower the rate. 

In such a situation, researchers search for effective and efficient motivators of EBF practices in recent years. Researchers conducted different studies in many settings and geographic contexts and came up with different motivators of EBF practices such as mothers’ knowledge and attitude, fathers’ attitude, previous breastfeeding experience, self-confidence [18,19,20,21,22] etc. However, fathers’ EBF knowledge, attitude, and support have rarely been studied in this regard, specifically in the case of Bangladesh. The rationale for considering Bangladesh as a sample country is that it is a small country that has gained a remarkable advancement in female education [23] in recent years. It is generally expected that higher educated mothers and fathers have better knowledge about the merits of EBF and a stronger attitude to EBF [24]. Moreover, the country has recently gained the status of a lower middle income economy by the World Bank [13]. 

This research study demonstrates several novel points. Firstly, it explores fathers’ factors in achieving EBF success. Secondly, it considers a widely accepted theoretical framework of theory of planned behavior (TPB). Thirdly, the research considers two different dimensions based on subsamples based on delivery mode. Fourthly, data for the research is collected for a nationally distributed sample which may represent the country. 

The research found that fathers’ knowledge undoubtedly assisted mothers’ EBF knowledge enrichment which result in a positive attitude of mothers to initiate and continue EBF. Moreover, fathers’ positive attitude to EBF practice by the mother and the support offered him to his wife boost her attitude as well. Thus a highly positive attitude translated into stronger intention to EBF which enhanced the chances of successful EBF practice. Additionally, the research found that perceived behavioral control is important for both sub-sample EBF practice while self-efficacy is found to be ineffective for cesarean delivery subsample. The previous breastfeeding experience is found to be inefficient for both sub-samples and the complete sample. The research also finds that control variables, income and profession is to a slight extent important determining EBF practice, however, residence and education level did not contribute to enrich EBF practices. 

## 2. The Theoretical Explanations and Hypothesis Development

### 2.1. Mothers Knowledge and Experience of Exclusive Breastfeeding (EBF)

Proper contextual knowledge can help individuals to have higher performance in practice, whereas inadequate knowledge definitely leads to poor performance. Researchers often acknowledged that changes in human attitude largely depends on knowledge and belief [25] of the concerned phenomena. In practicing EBF, mothers’ attitude is highly important, primarily explained by maternal knowledge [26]. Lack of positive attitudes to EBF backed by a low level of knowledge may cause low chance of timely initiation and continuation of EBF [27]. Moreover, education and previous experience may also be of an important source of knowledge [28] and form an attitude towards performance. In the case of breastfeeding, a multiparous mother’s prior breastfeeding experience can significantly affect her attitude toward EBF [29]. Thus, it was essential to investigate the impact of EBF knowledge and prior experience on mothers’ EBF attitudes.

**Hypotheses** **1 (H1).** *A mother’s knowledge of EBF has a significant impact on her attitude.*

**Hypotheses** **2 (H2).** *A mother’s prior experience of EBF significantly determines EBF attitude.*

### 2.2. Fathers’ Knowledge of EBF

Researchers explored mothers’ knowledge on child nutrition in various studies, while a few research works acknowledged the importance of father’s knowledge in this era [30]. Although breastfeeding or EBF is the role which is primarily borne by mothers [21], fathers with proper EBF knowledge can greatly support mothers [31] in practicing EBF. This is because a father with proper relevant knowledge can encourage his wife/partner and enrich her knowledge for breastfeeding initiation and continuation [20]. Moreover, a mother’s breastfeeding attitude is often influenced by the relevant knowledge depth of their accompanying adults [22], while the father is deemed to accompany the mother for a long period. Therefore, a fathers’ appropriate EBF knowledge is hypothesized as a support for his partner/wife to enrich EBF knowledge and attitude.

**Hypotheses** **3 (H3).** *A father’s knowledge of EBF positively influences a mother’s EBF knowledge.*

### 2.3. Fathers’ Attitude to Support EBF

An attitude towards some behavior is the result of knowledge about the matter [24]. While fathers have the most important role in accompanying mothers in parenthood and infant feeding [19,32], their knowledge and attitude are also vital in this regard. Attitude is a complex phenomenon where a father’s positive attitude towards EBF is largely dependent on proper knowledge on EBF [32], its necessity and benefits, potential losses for not practicing EBF, correct strategies of breastfeeding and so forth. Moreover, a father’s attitude as perceived by his partner/wife may shape her subjective (subjective norms are the perceived believes of other people who are important to the subject/individual about a specific behavior. For example, an individual’s thoughts about his/her family member’s willingness that he/she performs some activity) norms about EBF As a father is perceived to be the most important family role player [32]. Thus, fathers’ attitudes to support EBF can independently and significantly contribute to the perceived subjective norms of mothers. Hence, we hypothesize that:

**Hypotheses** **4 (H4).** *A father’s knowledge about breastfeeding form his attitude to support EBF.*

**Hypotheses** **5 (H5).** *A father’s attitude to support EBF can independently shape perceived subjective norms of a mother.*

### 2.4. Fathers’ Support towards EBF 

It is always important that a father is actively involved in breastfeeding activities to ensure mothers’ EBF intention [21] and duration as per guidelines [20,21]. A father can offer several kinds of support to his breastfeeding partner/wife such as acquiring and sharing EBF knowledge, directly helping her to be comfortable while breastfeeding, indirect instrumental support by supporting household and baby care activities, emotional supports by valuing and praising the breastfeeding partner for her efforts and endeavors, and easing himself from placing demands that may hamper breastfeeding [18,21,33]. Appropriate supports from the father can help the mother to have more rest, be comfortable and draw more concentration on breastfeeding the baby, which improves the overall breastfeeding duration and exclusivity [20]. To be more specific, the fathers’ proper knowledge, positive attitude to EBF and activities to support breastfeeding associated activities enhance a mothers’ attitudes positively towards breastfeeding the baby [21]. However, fathers’ supportive performance is often driven by their own attitude towards EBF [18]. A survey of literature by Sherriff, et al. [34] identified that a father’s support to EBF is formed by their won knowledge and attitude on EBF and may affect forms of offering their wives/partners decision-making support, practical physical and emotional support. Thus, it was plausible for us to investigate the influence of fathers’ attitude toward fathers’ support and fathers’ support activities to maternal attitude to EBF.

**Hypotheses** **6 (H6).** *Fathers possessing a higher EBF attitude are more prone to perform EBF supportive activities.*

**Hypotheses** **7 (H7).** *Father’s EBF supportive activities positively influence a mother’s attitude to EBF.*

### 2.5. Maternal Attitude to EBF

Attitude is a complex human behavioral phenomenon [32]. There are lots of factors influencing maternal attitude to EBF. The attitude a mother perceives towards breastfeeding her baby is perhaps the most important factor to determine her intention to breastfeed and feeding behavior. A positive maternal attitude towards breastfeeding can lead to early initiation, better frequency, and longer breastfeeding [35] and its success [36]. The mothers who possess a positive attitude to EBF are more likely to breastfeed their babies, whereas those with a negative attitude are less likely to breastfeed [2]. The chance that a baby will be breastfed as per the standard guideline mostly depends on the parental attitude perception and milk supply [37,38]. Therefore, as long as the proper supply is not an obstacle to initiate and continue breastfeeding, it is the mother’s attitude to EBF which determine her intention to or not to initiate and continue EBF.

**Hypotheses** **8 (H8).** *Maternal attitude towards EBF can positively determine her EBF intention.*

### 2.6. Subjective Norms

Subjective norms are the perception about other important persons’ believes and desires on a specific behavior or activity. In other words, this is termed as social pressure from the peer group or close people for any particular behavior. Subjective norms can formulate behavioral intention towards some specific behavior [39]. What other people, for example, intimate partner/husband, parents, close friends and relatives, closest neighbors, colleagues, doctors, nurses etc., think and suggest about infant feeding methods and strategies are very important to an expectant new mother. Peer group people’s views form a mothers’ subjective norms, which lead to intention to or not to breastfeed the baby exclusively [22], especially the norms received from the husband, family members and close peers have the most important power to drive mother’s intention to breastfeed [40].

**Hypotheses** **9 (H9).** *Mother’s subjective norms significantly influence her EBF intention.*

### 2.7. Perceived Behavioral Control (PBC)

The perceived ease of difficulty in performing certain behavior by an individual is termed as behavioral control [41]. The difficulty is assumed from different barriers such as internal barriers (i.e., the barriers from his/her lack of confidence, termed as self-efficacy) and external barriers created from many relevant factors i.e., perceived control [42,43]. The perceived ability to control difficulties is likely to positively add values to intention to perform by providing a measure of manageability of the barriers [44]. Perceived behavioral control (PBC) is the construct that differentiates TPB from the theory of reasoned action (TRA) [39,45]. A mother may have several short of external obstacles to practice EBF, such as sickness, unavailability of private places to breastfeed, getting back to school or work, having school or office not to be breastfeeding friendly (i.e., no availability of required time and place), long traveling to work or school etc. A mother’s belief that she can overcome these difficulties in a sound way and her personal confidence can build her intention to practice EBF. However, control behavior remained as an inconclusive predictor of breastfeeding intention in the literature [41] due to diversified findings of the relations. Therefore, the perceived control and self-efficacy may have a strong and direct association with the actual practice of EBF [41,46].

**Hypotheses** **10 (H10).** *Self-Efficacy has a positive impact on mother’s intention to practice EBF.*

**Hypotheses** **11 (H11).** *Perceived control has a positive impact on mother’s intention to practice EBF.*

**Hypotheses** **12 (H12).** *Self-Efficacy has a positive impact on mother’s practice of EBF.*

**Hypotheses** **13 (H13).** *Perceived control has a positive impact on mother’s practice of EBF.*

### 2.8. EBF Intention

An individual’s attitude to something, his/her perceived desire of peer group of people (subjective norms) and perceived level of difficulty or barriers determine his/her intention to perform the behavior [47]. The theory of reasoned action (TRA), considers behavioral intention to perform or not to perform a specific action as the best predictor of reasoned action. It is predicted by attitude and subjective norms, which lead to forming a behavioral intention. Ajzen [48] assumes that behavioral intention as just the intention to give a try to perform or refrain from an activity. However, Ajzen [39] explained the importance of perceived behavioral control to form one’s intention to behavior. Thus, it can be said that a stronger attitude, positive perception of peers, and confidence to overcome the difficulties or barriers of breastfeeding exclusively lead a mother’s EBF intention to turn into actual practice.

**Hypotheses** **14 (H14).** *EBF intention of a mother significantly predicts her practice of EBF.*

**Figure 1:**
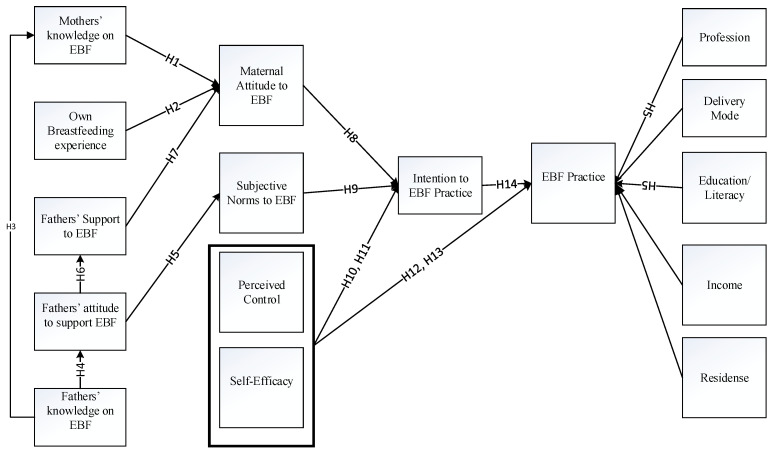
Father’s knowledge and attitude towards mothers’ practice of exclusive breastfeeding (EBF): the theoretical relationship in accordance with the theory of planned behavior (TPB).

Figure 1 presents the relations and the hypotheses.

## 3. Materials and Methods

### 3.1. Design

The study was based on an observational design that includes parents having the youngest children between 0–6 months of age. The study was designed to evaluate the role of paternal, knowledge and attitude, social peer pressures and control factors which can potentially influence maternal intention and practice to EBF. Finally, the impacts of variables were examined to reveal the practical reality existing in Bangladesh in regards to EBF within a TPB framework and with a structural equation model (SEM).

### 3.2. Settings

The study was conducted in a national representative sample of Bangladesh. The sample was taken from the divisional cities of the country. In Bangladesh, a division (administrative zone) is comprised of several districts and the headquarter of the division is the divisional city. Bangladesh currently has eight divisions namely, Dhaka, Chittagong, Khulna, Rajshahi, Rangpur, Sylhet, Barisal and Mymensingh, whereas Dhaka is the capital of the country as well. The reasons for considering city dwellers were (i) in Bangladesh cities have better educational and work concentration in comparison to suburban or rural areas, (ii) other infrastructures like mobile phone network and internet access are available in all cities but scarce in village areas (iii) people from the districts and the rural regions concentrate to the cities for education and jobs. Thus we expected divisional cities to be representative of the total geographical administrative division and hence sampling from all divisional cities will make the study representative of the country. 

### 3.3. Sampling

To determine the total sample size we employed the procedure proposed by Kothari and Garg [49] taking moderately educated females as target fraction and find that we should have at least 312 responses. The sample size is determined by dividing the multiplication result of the value of the standard variate, sample proportions, and population size with the summation of the multiplication results of the squared value of standard error and population size less 1, and the value of standard variate and sample proportions. To make the sample more representative, we firstly determined quota for each city which based on the ratio to required total sample and the current population of the city. The data regarding total population, birth, and education percentages come from World Bank’s World Development Indicators [50], while the data regarding population in each city under consideration came from World Population Review [51]. Taking 30% data loss into account, the sample size required was 405. Thus we came up with a estimated sample size of 242, 92, 31, 16, 8, 6, 5 and 5 for Dhaka, Chittagong, Khulna, Rajshahi, Rangpur, Sylhet, Barisal and Mymensingh respectively. Secondly, we apply convenience sampling technique to invite parents as potential respondents. Convenience sampling is one of non-probability sampling methods where the participants are selected based on their accessibility [52] in a non-random fashion. This technique is very useful to undertake in a quick, low expensive way and can foster target community engagement in the study. It often utilizes snowball technique using word of mouth and social media [53]. To recruit the sample, we published our invitation to volunteer our research via social media such as Facebook mentioning key inclusion criteria and receive 130 primary responses and collected data about their demographics, current living status and the data relevant to our inclusion and exclusion criteria. After excluding non-eligible respondents, we recruited a total of 44 volunteers only. Then we contacted them over phone and shared the inclusion and exclusion specifications of the study and requested them to invite parents from their acquaintance who matched the criteria. Thus we invited a total 954 parental cases to respond to the questionnaire.

The convenience sampling technique may sometimes cause selection bias. However, in our case, recruitment of volunteers via media at the first stage and then via snowball technique, restricted to apply researchers’ choice and ease selection bias. Moreover, the applied quota technique allows the sample represent the country and reduced the potential of selection bias. The study remained unrestricted in respect of the sociodemographic variation of parents i.e., the study accepted equally parental cases from different levels of sociodemographic characteristics such as income level, age, education, race/religion, number of children, husbands’ profession and so, which also helped to reduce the chance of selection bias.

### 3.4. Inclusion Criteria and Exclusion Criteria 

Several inclusion criteria had been followed while selecting the respondents such as (i) fathers and mothers were of at least 18 years of age and of sound mental (not suffering from lunacy, insanity or idiocy) and physical health (not having any physical incapacity easing the potential of the mother to practice EBF and/or the father to support her) who had at least one child aged between 0 to 6 months. Unsound mothers and fathers would have little chance to practice and support EBF; (ii) fathers and mothers had to be capable of reading and listening English and/or Bengali (native language of Bangladesh); (iii) parents should have communication devices (e.g., mobile phone) and either full-time or optional access to the internet (such choice was backed by two major reasons. Firstly, Bangladesh is a Muslim country and culturally conservative enough [54]. Bangladeshi women and mothers might not feel free to conduct a face to face interview on an issue like breastfeeding. Secondly, the survey was conducted during April to June of 2020 while the outbreak of coronavirus disease 2019 (COVID-19) was a big barrier for a face to face interview. However, more than 90% households in the country own communication devices, mostly mobile phones [55]. Moreover, most of the people already have access to internet [56], especially city dwellers. Therefore, there would have been little chance of a communication barrier and selection bias); (iv) mothers who initiated early breastfeeding; (v) parents who lived together at the same residence with their child/children as who live separately, will have little chance to support. The study excluded (i) high-risk infant cases with chronic illness and other complications including anemia, low birth weight, congenital anomalies (for example, cleft palate or cleft lips), etc. as EBF might be hampered due to illness (ii) fathers and mothers with previous mental (for example, generalized anxiety disorder (GAD), social anxiety disorder (SAD), common mental disorders (CMD)) and physical disorders which might have restricted initiation and continuation of breastfeeding; (iii) fathers/mothers who were separated or divorced before or after childbirth as that father will have no chance to support his ex-wife; and (iv) mothers and/or infants who had any other unfavorable properties to be breastfed or to be supported by a father to breastfeed.

### 3.5. Measurements

This research considered fathers and mothers as separate entities and surveyed fathers and mothers independently to assess mother’s knowledge and attitude which builds the intention to practice EBF and father’s attitude to support mothers in practicing EBF. Questionnaires were used both for fathers and mothers separately. Both of the questionnaires were divided into two parts—the first part contained 11-question demographic information. The second part contained key measurement questions relevant to breastfeeding for mothers and breastfeeding support offered by fathers to the mothers. We relied on recent literature for example [57,58] to design the demographic information consisting of current age, education level, personal income, delivery mode for the youngest child, maternity leave, number of children and age of youngest child, region (city) of residence and contact details.

#### 3.5.1. Exclusive Breastfeeding and Practices of EBF 

As per WHO [59], exclusive breastfeeding is termed as giving only breastmilk to the baby from the first hour of birth and no other food or liquid except necessary medication, up to the age of six months. To assess the practices of EBF of mothers we largely follow Emmanuel and Clow [60]. This part of the questionnaire contains twelve questions including mother’s self-reports assessed questions about exclusive breastfeeding practices by asking their initiation at the first hour and continued practices at one month, three months and six months of the baby’s age. Questions about pre-plans of EBF practice and feeding after six months up to two years were also included. We followed the same style of questioning for the fathers to assess their plans and activities to support the mother for practicing EBF. The questions are in statement form which give multiple choice answering options with a five-point Likert scale consisting of 1 as strongly disagree to 5 as strongly agree.

#### 3.5.2. Intention to Practice EBF

In the present study, we measured the intention of mothers to exclusively breastfeed the baby as per standard guidelines (from 0–6 months) with the Infant Feeding Intentions scale (IFI) following Nommsen-Rivers and Dewey [61]. The IFI scale deals with a total of five items whereas the first two items deal with the strength of intentions of mothers to breastfeed and the rest of the items deal with the strength of their willingness to continue exclusive breastfeeding at one, three and six months of age.

#### 3.5.3. EBF Attitude

To explore EBF attitude of mothers and fathers, we applied the “Iowa Infant Feeding Attitude Scale” (IIFAS) developed by Mora, et al. [62]. This scale is an easily administered scale to reveal the feeding attitude. At the development stage, this scale was applied in three studies by Mora, Russell, Dungy, Losch and Dusdieker [62] and found to be a valid and reliable instrument with Cronbach’s α ranging from 0.68 to 0.86. The instrument can predict the feeding method choice. The scale is a combination of 17 items out of which 9 are reverse coded items. We adapted the questionnaire with similar language to the original and a 5-point Likert scale to assess the attitude of mothers to breastfeed their babies exclusively. Moreover, to assess the attitude of fathers to support their partners for EBF, we asked a similar set of questions.

#### 3.5.4. Breastfeeding Knowledge and Experience

We followed Osibogun, Olufunlayo and Oyibo [46] to prepare a knowledge assessment tool. We developed 11-item tool/questionnaire containing statements about different aspects of EBF of which the first one assessed the engagement of the interviewee (father/mother) in breastfeeding talks, i.e., how frequently he/she used to hear about EBF. The subsequent five items assess the knowledge about what EBF is and how it benefits. Items 7–9 assessed knowledge about the expected benefit of EBF up to the first six months of the baby regarding producing energy and prevention from diseases. The next item assessed knowledge about timing of feeding breastmilk. The last item dealt with knowledge of the importance of EBF in comparison to formula feeding. Out of the 11 items, three were reverse coded. We followed the same methodology to assess the knowledge of fathers about EBF. The experience variable is measured about the mother only with a single item construct which is a binary variable i.e., 0 for primiparous mothers and 1 for multiparous mothers. This is done with the belief that multiparous mothers had a prior chance to breastfeed elder children.

#### 3.5.5. Normative Beliefs

We use the TPB guided framework following Giles, Connor, McClenahan, Mallett, Stewart-Knox and Wright [43] to formulate items for subjective norm construct. The construct contained six items that asked about the perception of people who are important to mother, mother’s mother, family members, husband/partner, doctor and nurse, close friends and colleague about EBF. The items directly asked the surveyed mothers about their beliefs on how the others important peers wanted them to feed their babies (for example “My mother would like me to breastfeed my baby exclusively”). It is noteworthy that this construct was applied for mothers only as this is about their normative beliefs regarding EBF and not about that of fathers’.

#### 3.5.6. Perceived Behavioral Control

Perceived behavioral control (PBC) is determined by perceived ease of difficulty to perform certain behavior. Such control believes or perceived behavioral control constitutes internal and external control factors. The first factor, internal control, emphasizes how a person assumes him/herself to behave while performing some activity. The internal control, termed as self-efficacy, depends largely on the actor’s knowledge, skill, and ability to perform the activity or self-confidence in some activities. On the other hand, external control is the set of factors that control one’s behavioral intention and/or actual behavior due to external factors. One’s family members, friends, colleagues and peers often shape one’s behavioral intention or behavior. We follow Ajzen [39] and Giles, Connor, McClenahan, Mallett, Stewart-Knox and Wright [43] to formulate questions for control beliefs. This part of the questionnaire contains 11 questions while three of them represent self-efficacy and the remaining eight represent perceived behavioral control. The self-efficacy part asks three questions about the respondent’s self confidence regarding her internal factors to perform EBF, while PBC part asks seven questions about her self confidence regarding the external factors, such as her confidence to overcome EBF obstacles raised by external affairs. This construct is targeted only to mothers as they perform the primary responsibility to breastfeed their babies.

## 4. Data Collection, Management and Ethical Considerations 

Before the final survey, we conducted a pilot survey on a small sample of 30 participants (mothers and fathers) to check the readability and validity of our questionnaire. We found a necessity for minor revision to enhance its readability and did the revisions accordingly. Moreover, we added translations into Bengali (the language of Bangladesh) of the questions/statements in brackets which were placed adjacent to each sentence/question/answering option. The translation was primarily done by the corresponding author and verified and modified by a professor of Bengali language and literature, Jatiya Kabi Kazi Nazrul, at Islam University, Mymensingh, Bangladesh. A total of 954 pairs of questionnaires were distributed considering parental cases that match our inclusion and exclusion criteria while keeping in mind the quota for each city. We went for such extensive invitation of respondents due to the low response rate (40–50%) by the online survey [63] which is identified as very low in health care studies [64,65]. As described in Section 3.3 and Section 3.4, the respondents were invited to volunteer the research firstly by social and news media and secondly by extending the invitation through snowballing technique undertaken via primarily invited respondents according to their convenience. The data collection tool (questionnaire) was prepared using an online form employing ‘Google forms’. The links of the forms were shared to the prospective respondents by the investigators and primarily selected volunteers through online and social media (e.g., Facebook, WeChat, personal emails). At the beginning of the response, the respondents first would find a consent form which they had to respond by yes/no options. If they expressed their consent by selecting “yes”, they could get forward to respond demographic information questions. Upon completion of the demographic information they could see and respond to questions regarding EBF. The participants were able to contact us via email and telephone at any time to ask any question. To maintain the highest level of ethical standards while conducting the research, we followed several steps. Firstly, the respondents responded only on their option to do. Secondly, they could withdraw their response at any point during the study. Thirdly, the confidentiality of their responses were maintained and the data was used only for research purposes. Fourthly, inference is drawn only based upon the findings revealed in standard procedure. A total of 398 pairs of responses were received (single responses were not considered) which was around 42% of the total questionnaires sent. However, the minimum number required for our study was met even after the exclusion of incomplete responses. After the exclusion of invalid and incomplete observations, (inaccurate, duplicate, irrelevant and incomplete observations were removed from the dataset) we were able to retain 332 responses for analysis. While constructing the sample all dimensionalities (e.g., social class) were treated equally, i.e., parental cases from each dimension had the equal chance to be included, despite the issue of convenience sampling. Furthermore, from demographic analysis (presented in Section 6) we found that participants form all age, profession, income, income, residence, religion and education groups were included in the sample.

## 5. Data Analysis

### Evaluation of Multivariate Assumptions

After we obtained the data, first, we analyzed the demographic characteristics of participants. Then we reversed the reverse coded items with standard procedure and performed several pre-estimation tests to confirm the readiness and appropriateness of the data to verify the readiness for multivariate analysis such as multicollinearity test by using correlation and variance influence factor (VIF). The correlation coefficient between constructs lower than 0.90 [66] and the VIF of below 10 [67] represent low potential risk of multicollinearity. However, any construct gaining VIF values greater than 3.3 is at risk of common method bias (a phenomenon usually caused by SEM measurement method and not by relational effects of the variables under study) [68] which represents spurious correlation among variables and may lead to erroneous conclusions [69], especially caused by type II error [70]. 

To assess the validity and reliability we assessed measurement model [71] which includes reliability measures such as, Cronbach’s alpha and Dijkstra-Henseler’s rho; convergent validity measures such as, item loadings, composite reliability and average variance extracted (AVE) and discriminant validity measures such as the Fornell–Larcker criterion and Heterotrait–Monotrait ratio. After we found the data fit for multivariate analysis, and the constructs as valid and reliable, we were reasonably assured about the absence of multicollinearity to pursue a structural equation model (SEM) path analysis which was in the present case designed in accordance with the TPB framework. We used a variance-based multi-group SEM to simultaneously investigate a series of linear relationships [72] between different factors in EBF. This approach of SEM is capable enough of dealing with different stages of constructs at the same time while other types of analysis modules, such as multiple regression, are inappropriate and have limited capacity in this regard [73]. The path coefficients driven by SEM show us the strength of association in each relationship while the significance is measured by *t* statistics and/or *p* values.

## 6. Results 

### 6.1. The Characteristics of Participant/Couples

We analyzed the demographic characteristics of i.e., fathers and mothers. However, as breastfeeding is the responsibility primarily borne by mothers, we emphasized their characteristics. We considered the scenario according to the delivery mode of the last birth in addition to the total parental demographic characteristics. We found that respondents from all social categories were included in the survey, despite variations in percentage of participation, which was common in health literature [74,75]. Among 332 mothers 121 (36.45%) had to undergo a cesarean section while the rest (63.55%) had a vaginal delivery of their youngest children. As the samples were taken from different cities in ratio to the total population currently dwelling in the concerned city, the number of samples varied much among the cities having the highest percentage from Dhaka while the lowest was from Barisal. In terms of religion the highest fraction came from the Muslims (82.22%) while the other were Christians, Buddhists and Hindus; 67.17% of the total mothers surveyed were employed while the rest were were searching for jobs and/or housewives. In terms of education, more than a half (51.81%) of the total mothers completed a master’s degree while 22.29%, 19.88% of the total achieved only a bachelor and secondary school degrees respectively, while the rest had below secondary level education. We followed Bhowmik, Biswas and Woldegiorgis [74] and current salary structures practiced in Bangladesh, to categorize the economic situation of fathers and mothers as ‘poorest’, ‘poorer’, ‘middle’, ‘richer’ and ‘richest’ based upon their monthly income of lower than $100, $100–$300, $300–$500, $500–$800 and above $800, respectively. As per the monthly income of the participants 113 (34.04%) had a very low (less than $100) or no income, 38 (11.45%) had moderate income of $100–$300, 106 (31.93%) had good income ($300–$500), 56 (16.87%) had satisfactory income ($500–$800), while only 17 (5.12) had a high income (>$800). As the matter of EBF intension and practice might vary in case of mothers having a vaginal and cesarean delivery modes, it was important to look into these phenomena. Thus we considered to have an overview of mothers’ characteristics in accordance to latest delivery mode which is presented here in Table 1. In respect to respondent fathers we found majority of them were either employed and/or engaged in businesses (97.6%). This may be due to the fact that in Bangladesh men are supposed to bear the major responsibility of family expenditure. Hence most of the males in Bangladesh rarely think about getting married before they are employed or have established a business. The formal education scenario of fathers was such that, 10.2%, 3.3%, 29.2% and 57.2% of them had lower than secondary, secondary, bachelor and master’s degree education. As per income category, 14.2%, 17.5%, 30.4%, 31.6% and 6.3% of the responding fathers were from ‘poorest’, ‘poorer’, ‘middle’, ‘richer’ and ‘richest’ categories respectively. However, to maintain brevity, we avoid to present the parental demographic characteristics in a ratio to total respondents here and present in Table A3 in Appendix A. 

#### List of Variables

We considered a number of variables in this study. To help the reader, the variables along with their acronyms are listed below in Table 2.

### 6.2. Evaluation of Multivariate Assumptions

Before analysis of the data for the hypothesized relations, we checked the fitness of our data to meet multivariate assumptions [76] which in other sense can be acknowledged as preliminary diagnostics. We used the Fornell–Larcker criterion and VIF, the results of which are presented below. 

Multicollinearity is a serious issue which adversely may affect analysis results lead to spurious inferences. Table 3 presents correlation coefficients from Fornell-Larcker criterion. All values in the table were below 0.90, which meant that there was no significant chance of multicollinearity [66]. Therefore, the variables and data under consideration gained confidence for multivariate analysis. However, to confirm this we additionally conducted VIF analysis which is presented in Table A4 in Appendix A. VIF analysis can also estimate the chance of multicollinearity. The factors for each variable should be below 3.3 [67,68] to confirm the variables free form multicollinearity. We found VIF of variables to be within the range of 1.0 to 1.838, which are below the recommended values and thus, we had confidence that our data and variables were free from potential risks of multicollinearity and common method bias and could be used for multivariate analysis.

### 6.3. Measurement Model

We used the measures that are frequently used in literature to assess the internal reliability, convergent and discriminant validity of our model. We used common methods to measure internal reliability such as Cronbach’s alpha and Dijkstra-Henseler’s rho (ρA). We confirmed convergent validity by checking the item loadings, composite reliability and average variance extracted (AVE). We measured discriminant validity with the Fornell–Larcker criterion-based correlation [77,78]. The criteria measured the degree of differences among the overlapping constructs. An important condition of the Fornell–Larcker criterion is that the square root of AVE for a variable should be higher than the correlation of that variable with others. Additionally, we utilized an alternative discriminant validity measure, the Heterotrait–Monotrait ratio (HTMT ratio) as developed by Henseler, et al. [79], to measure discriminant validity. The results of mentioned criteria are presented in facing tables.

In the present study, the condition is met well as can be seen in Table 2. 

Table 3 shows that the reliability criteria, i.e., Cronbach’s alpha and Dijkstra-Henseler’s rho (ρA) both were desirably having higher value over a suggested value of 0.70 [80]. This meant that the constructs were reliable enough in terms of consistency which indicates that the tool used for data collection (the questionnaire) was reliable. The convergent validity was ensured by screening the item loading in column 2 composite reliability in column 5 and average variance extracted (AVE) in column 6 of Table 4. The calculated values of item loading were found to be over 0.50 [72] whereas the composite reliability for every variable was above the recommended minimum value 0.70 [80]. The AVE for every variable was above the correlation of that variable with other variables (Table 2) and above the recommended minimum value of 0.50 [77], which was desirable. It meant that theoretically connected constructs were found to be correlated which happens, for example, when more than one similar question reveals similar results.

Table 5 presents the measure of discriminant validity, HTMT ratio. We found a significantly below 1.00 HTMT score which reveals the discriminant validity of the variables [77]. More specifically, HTMT score shows better validity if the values are below 0.85 [81]. For the present study, we found that that all values were below the threshold of 0.85. Thus it was confirmed that non-overlapping factors were not overlapped.

As both of convergent and discriminant validity was confirmed about our constructs, our constructs were valid and were eligible for further use.

### 6.4. The Structural Model

We hypothesized three SEMs. The first one analyzed Bangladeshi fathers’ knowledge, attitude and intended support to mothers for practicing EBF based on our complete sample Table 6. The second and third SEMs split the sample into dimensions based on delivery mode and analyzed the similar fact with a comparative attitude. However, the results from second and third SEMs are not included here to maintain brevity and are presented in Table A1 and Table A2 in Appendix A. 

The SEMs find that fathers’ knowledge about EBF has a significant positive impact on maternal EBF knowledge (*β* = 0.845–0.863); fathers’ support to the mothers to practice EBF has a significant positive impact on maternal attitude (*β* = 0.881–0.910). On the other hand mothers’ knowledge has a significant positive role in boosting mothers’ EBF attitudes (*β* = 0.490–0.820). Mothers’ positive attitude is found to gain a significant role from mothers’ intention (*β* = 0.142–0.524) which in turn leads to higher practice of EBF (*β* = 0.030–0.243). The study also considers other important factors and demographic variables which formulate maternal attitude to practice EBF and enhance EBF practices. The other factors and variables include, for example, mothers’ previous breastfeeding experiences (if any), subjective norms and perceived behavioral control. Moreover, the demographic variables considered were the profession of mother, educational level, household income, and residence. These variables were found to have a varying role across the SEMs. 

## 7. Discussion

**Figure 2:**
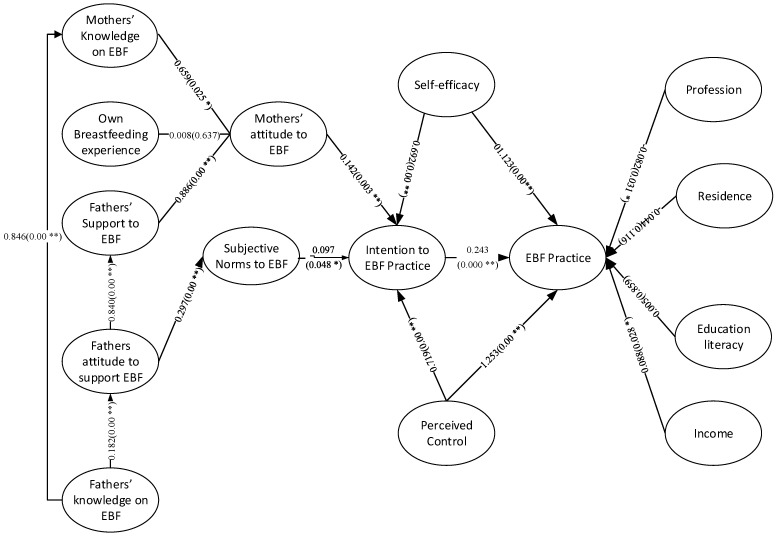
SEM analysis on father’s knowledge and attitude on mother’s EBF knowledge, attitude and practice in Bangladesh (on complete sample). * Significant at *p* < 0.05; ** Significant at *p* < 0.01.

**Figure 3:**
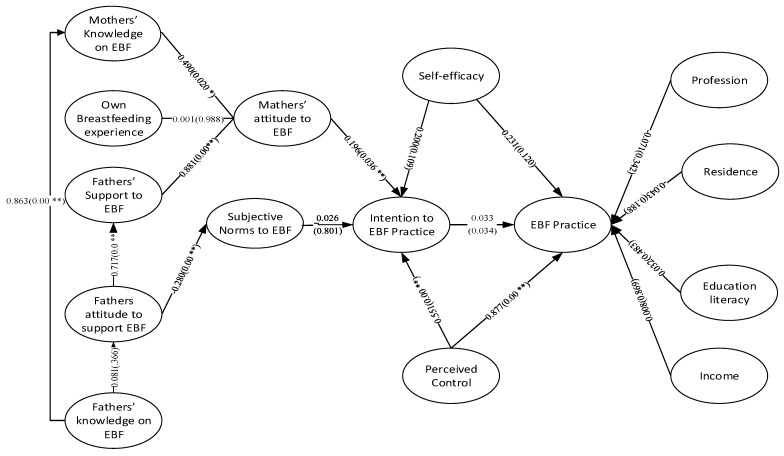
Multi group analyzing the role of father’s knowledge and attitude on mother’s BF knowledge, attitude and practice in Bangladesh (on cesarean delivery subsample). * Significant at *p* < 0.05; ** Significant at *p* < 0.01.

**Figure 4:**
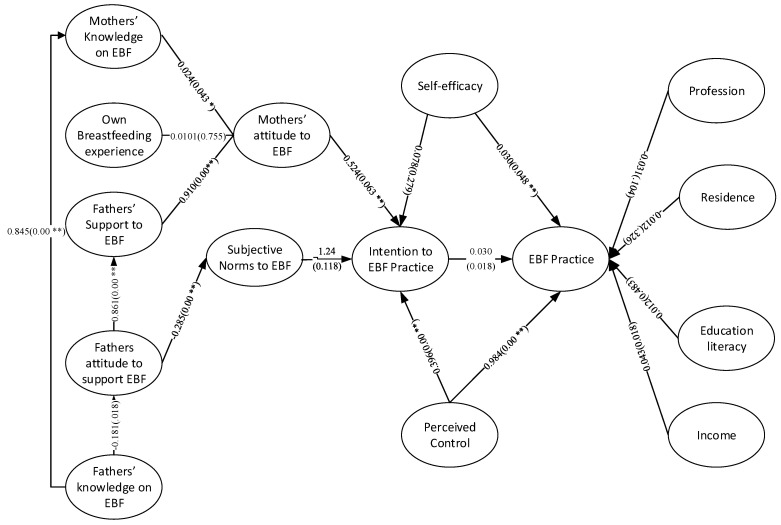
Multi group analyzing the role of father’s knowledge and attitude on mother’s breastfeeding (BF) knowledge, attitude and practice in Bangladesh (on vaginal delivery subsample). * Significant at *p* < 0.05; ** Significant at *p* < 0.01.

The study, to the best of our knowledge, was the first ever to diagnose the role of fathers’ knowledge about and attitude to support EBF on forming mothers’ knowledge, attitude and practice of EBF based on multi-group SEM. To test the broad aspects, we followed TPB to formulate our research framework that guides us to test Bangladeshi fathers’ knowledge of EBF and attitude to support mothers in practicing EBF on mothers’ knowledge, attitude and practice of EBF. To investigate the facts and figures, following TPB the SEMs relied on a number of principal hypotheses which direct the relationships of fathers’ knowledge and support offered to mothers and other relevant constructs to, finally, practice of EBF by the mothers. The facts revealed are presented in Figure 2, Figure 3 and Figure 4 here below for the complete sample, cesarean delivery cases sub-sample and vaginal delivery cases sub-sample, respectively.

As generally known, any support by knowledge sharing is the greatest support for the recipient entity. That is, if a father can achieve knowledge about EBF, its necessity and strategies and can enlighten his partner i.e., the mother with his achieved knowledge. It is noteworthy that our study found fathers’ knowledge to explain more than 80% of mothers’ knowledge about EBF as we found the coefficient values between these two variables as 0.846 (complete sample); 0.863 (cesarean delivery sub-sample) and 0.845 (vaginal delivery sub-sample). The highly significant coefficient explains the high importance of fathers’ role play in this era. This finding is consistent with Rempel, Rempel and Moore [21] and Rempel and Rempel [33] as the authors suggested that a father can equip a mother with proper EBF knowledge. 

On the other hand, our findings suggest that fathers’ breastfeeding knowledge enrich his attitude to offer support for EBF to the mother and consequently the enhanced positives attitude result in practical support offered by a father to his partner [18,39]. A father’s positive and stronger attitude is also found to directly and positively influence mother’s attitude [19,21,24] and mother’s subjective norms [32]. The maternal attitude to practice EBF, subjective norms and perceived behavioral control is found to significantly determine maternal intention to practice EBF [22,35,41,46,82] whereas the self-efficacy sub-component is found to be of less importance and indirectly determining maternal intention. 

Maternal intention being determined by maternal attitude, subjective norms, and perceived behavioral control to EBF, significantly determines actual practice of breastfeeding exclusively. The perceived behavioral control can also directly affect maternal practice of breastfeeding as the perceived behavioral control variable (jointly, self-efficacy and perceived control) is found to have a highly significant and positive coefficient. 

However, the demographic instances are not found to have much specific effect on EBF practices. More specifically, however, on the complete sample the variables like profession and income had a significant effect on EBF practices while place or city of residence of mothers and level of education showed insignificant relations. Coming to separated subsamples based on delivery mode we found that all four control variables showed insignificant effects in the case of cesarean delivery subsample and for the normal delivery subsample only income showed some significant and positive relation.

In summary, our findings reveal that for Bangladeshi mothers to practice EBF, her partner’s knowledge, attitude and support are significantly important. Once a father is well educated and knowledgeable about EBF, he can share his knowledge to his wife i.e., the mother, which can enrich EBF practice through enrichment of intention via an enhanced positive attitude towards EBF. The findings are mostly robust in the complete sample and cesarean and normal delivery subsamples. The tables containing path coefficients for the complete sample is presented in Table 5. However, for brevity path coefficients for subsamples of parental cases based on latest delivery mode of mothers—cesarean and vaginal delivery—are presented in Table A1 and Table A2 in Appendix A respectively. 

## 8. Practical Implications of the Research

Our study has some important practical implications. It reveals that in both vaginal and cesarean delivery cases, mothers’ knowledge and attitude to practice EBF is highly affected by that of fathers. Mothers’ attitude towards EBF significantly determines their intention to practice EBF as per guidelines. However, the scenario may vary across delivery mode. It has been revealed that Bangladeshi mothers’ attitude to EBF is not much influential in determining the intention in case of mothers who had cesarean delivery whereas the same is significantly influenced in the cases of normal delivery. This may be caused by mothers’ illness subsequent to cesarean section which resist positive attitudes to strong intention to practice EBF. Cesarean section usually delays the initiation of breastfeeding [83] which often results in cessation from EBF or breastfeeding as a whole. Thus health workers should be careful about the delivery mode. They should train more carefully about breastfeeding pre-partum and postpartum for planned cesarean delivery cases. We further find that perceived behavioral control has a significant effect on mothers’ EBF intention and EBF practice in complete sample, cesarean and normal delivery sub-samples. Moreover, the demographic elements like profession and income are found to be important; residence and level of education are not so important when considering the complete sample. While considering the subsamples the demographics become mostly insignificant. These have important implications for the policy makers and health workers. They should carefully design EBF promotional activities so as to enhance the maximum output. Moreover, as profession, residence, educational and income levels seem not to matter as much for either of the delivery modes, the care leaders should formulate EBF promotional policies for each group regardless of their profession, residence, educational and income levels. 

## 9. Limitations of the Study

Despite of several novel points, the study is not fully free from limitations. The study generalizes the finding based of the results driven. However, while concluding the research the limitations should be considered. Firstly, the study uses a multigroup SEM approach based on a TPB framework. The multigroup is constructed based on delivery mode which did not consider any other potential groups. Secondly, the usage of convenience sampling method may raise the potential of bias, which in turn, may weaken the generalizability of the study. Thirdly, the study considers previous pregnancy for multiparus mothers as only the direct source of breastfeeding experience and disregards all other potential indirect experience sources. This might have limited the measurement of breastfeeding experience. Fourthly, the study is based on a single country context, which although it is able to make very specific recommendations may not be generalizable to other countries. Finally, up to six months EBF practice has been measured as a self-reported outcome variable of the study. We have not monitored the initiation and continuation of EBF over time after the baby’s birth up to a prescribed time. 

## 10. Conclusions

This paper empirically investigates the roles of a mother in practicing exclusive breastfeeding (EBF) and father role in supporting the mother within a framework of the theory of planned behavior and with structural equation modeling. To investigate the issue, we consider Bangladesh, a lower-middle-income country, as a case considering a high fluctuation in the EBF rate prevailing in the country. We endeavor to represent the whole country and conducted a survey in eight divisional cities while each represents a region/division of the country. The survey was undertaken by sending online questionnaire forms to the identified parents while the parents are identified on a joint basis of quota and convenience sampling. A total of 954 parental cases were identified and provided a questionnaire for the survey. However, only 398 questionnaires were returned. After screening and shorting out incomplete or irrationally complete responses, 332 responses were included for analysis purposes. 

Analysis of data reveals that a father’s knowledge of EBF is of high importance in strengthening mother’s EBF knowledge and subjective norms in one way while in another way for enhancing the chances of other types of support (emotional and domestic) to be offered to a partner (i.e., the mother) via enhancing his attitude to support EBF. In line with the postulates of the theory of planned behavior, we also find that stronger maternal attitude to EBF, EBF supportive social norms and ease of behavioral control shape a mother’s strength of intention to breastfeed her baby exclusively, while we find that the higher the intention the better is the practice of EBF. Our findings are robust across different methodologies such SEM path analysis. These findings are important for relevant health policymakers. They should formulate and execute proper policies that encourage the health service providers to educate new and expectant fathers about EBF. Likewise, policies also may be adapted to encourage new and expectant fathers to encourage learning about EBF. Moreover, policymakers should formulate and implement policies that create social norms and ease perceived difficulties in practicing EBF, for example., For working and student mothers, proper maternity leave in all types of organization, daycare, and breastfeeding spaces in all types of schools and organizations. Community hospitals, educational institutes, formal and informal social media, and health educators may take responsibility for knowledge enhancement and awareness building by realizing the seriousness of this issue.

## Figures and Tables

**Table 1:** Mothers’ demographic characteristics in respect to delivery mode (N = 332).

Variables		Delivery Mode		*p*-Value
		Cesarean Delivery *n* = 121 *n* (%)	Vaginal Delivery *n* = 211 *n* (%)	
Age	18–25 Years	5 (41.7%)	7 (58.3%)	0.425
	26–33 Years	59 (34.3%)	113 (65.7%)	
	34–41 Years	56 (38.9%)	88 (61.1%)	
	42–49 Years	1 (25.0%)	3 (75.0%)	
Number of Living Children	1 Children	83 (35.8%)	149 (6.2%)	0.547
	2 Children’s	31 (39.2%)	48 (60.8%)	
	3 Children’s	7 (36.8%)	12 (63.2%)	
	4 Children’s	0 (0.0%)	2 (100.0%)	
Resident of the Division	Sylhet	3 (37.5%)	5 (62.5%)	0.290
	Mymensingh	1 (16.7%)	5 (83.3%)	
	Khulna	14 (41.2%)	20 (58.8%)	
	Rangpur	3 (30.0%)	7 (70.0%)	
	Chittagong	18 (40.0%)	27 (60.0%)	
	Barisal	0 (0.0%)	7 (100.0%)	
	Rajshahi	8 (42.1%)	11 (57.9%)	
	Dhaka	74 (36.5%)	129 (63.5%)	
Religion	Christians	3 (25.0%)	9 (75.0%)	0.683
	Buddhism	1 (25.0%)	3 (75.0%)	
	Hindu	18 (41.9%)	25 (58.1%)	
	Islam	99 (36.3%)	174 (63.7%)	
Profession	Searching for work	11 (33.3%)	22 (66.7%)	0.947
	Housewife	28 (36.8%)	48 (63.2%)	
	Public Sector Employee	41 (35.3%)	75 (64.7%)	
	Corporate Employee	41 (38.3%)	66 (61.7%)	
Education	Less than high school degree (Less than SSC)	9 (45.0%)	11 (55.0%)	0.699
	High school degree (HSC)	21 (31.8%)	45 (68.2%)	
	Bachelor degree	26 (35.1%)	48 (64.9%)	
	Masters’ degree and above	65 (37.8%)	107 (62.2%)	
Income Per Month	<$100	41 (36.3%)	72 (63.7%)	0.410
	$100–$300	18 (47.4%)	20 (52.6%)	
	$300–$500	38 (35.8%)	68 (64.2%)	
	$500–$800	20 (35.7%)	36 (64.3%)	
	>$800	4 (23.5%)	13 (76.5%)	

**Table 2:** List of variables along with abbreviation.

F.Know	Fathers’ Knowledge on EBF	M.Int	Mothers’ Intention to EBF Practice
F.Att	Fathers’ Attitude to Support EBF	M. Att	Mother’s attitude to EBF
F.Supp	Fathers’ Support to EBF	M.PC	Mothers’ Perceived Control
M.Know	Mothers’ Knowledge on EBF	M.SN	Mothers’ subjective norms
M.Exp	Mothers’ Own Breastfeeding Experience	M.SE	Mothers’ Self-Efficacy
EBF	EBF Practice		

**Table 3:** Correlation coefficient of variables in accordance with Fornell–Larcker criterion.

Constructs	F.Know	F.Att	F.Supp	M.Know	M.Exp	EBF	M.Int	M.Att	M.PC	M.SN	M.SE
F.Know	0.817										
F.Att	−0.181	0.762									
F.Supp	0.419	−0.109	0.789								
M.Know	0.711	−0.226	0.268	0.830							
M.Exp	0.063	0.070	0.057	0.018	1.000						
EBF	0.339	−0.224	0.243	0.368	0.011	0.759					
M.Int	0.124	−0.122	0.311	0.191	−0.056	0.401	0.796				
M.Att	−0.148	0.370	−0.107	−0.101	0.011	−0.157	0.385	0.772			
M.PC	0.346	−0.248	0.237	0.416	0.005	0.821	0.415	−0.149	0.768		
M.SN	0.500	−0.355	0.348	0.607	0.068	0.253	0.035	−0.290	0.292	0.799	
M.SE	−0.181	−0.120	−0.044	0.062	0.009	−0.118	−0.158	−0.202	−0.090	0.486	0.808

Note: Please see Table 2 for variable name elaborations.

**Table 4:** Results of reliability and convergent validity measures.

Constructs	Item Loading Range	Cronbach’s Alpha	rho_A	Composite Reliability	AVE
F.Know	0.763–0.857	0.929	0.931	0.941	0.668
F.Att	0.722–0.799	0.952	0.953	0.957	0.581
F.Supp	0.722–0.842	0.853	0.881	0.892	0.623
M.Know	0.761–0.889	0.935	0.938	0.946	0.689
M.Exp	1.000–1.000	1.000	1.000	1.000	1.000
EBF	0.707–0.817	0.918	0.920	0.931	0.576
M.Int	0.754–0.84	0.855	0.858	0.896	0.634
M.Att	0.726–0.814	0.955	0.955	0.959	0.596
M.PC	0.722–0.817	0.900	0.902	0.920	0.590
M.SN	0.772–0.824	0.887	0.889	0.914	0.639
M.SE	0.773–0.866	0.735	0.755	0.849	0.653

Note: Please see Table 2 for variable names elaboration: AVE = average variance extracted.

**Table 5:** Output of Heterotrait–Monotrait (HTMT) analysis.

Constructs	F.Know	F.Att	F.Supp	M.Know	M.Exp	EBF	M.Int	M.Att	M.PC	M.SN	M.SE
F.Know											
F.Att	0.193										
F.Supp	0.465	0.114									
M.Know	0.761	0.239	0.295								
M.Exp	0.067	0.088	0.068	0.047							
EBF	0.363	0.237	0.271	0.390	0.044						
M.Int	0.169	0.148	0.361	0.237	0.061	0.452					
M.Att	0.170	0.385	0.115	0.149	0.048	0.171	0.425				
M.PC	0.375	0.269	0.265	0.452	0.045	1.082	0.472	0.165			
M.SN	0.547	0.386	0.393	0.661	0.072	0.276	0.128	0.314	0.323		
M.SE	0.220	0.142	0.076	0.090	0.025	0.147	0.196	0.243	0.112	0.592	

Note: Please see Table 2 for variable names elaboration.

**Table 6:** Path coefficients of structural equation modelling (SEM) (using complete sample).

Hypotheses	Path	β	SD	*t*-Value	*p*-Value
H1	M.Know->M. Att	0.659	0.016	1.152	0.025
H2	M.Exp->M.Att	0.008	0.018	0.472	0.637
H3	F.Know->M.Know	0.846	0.013	63.446	0.000
H4	F.Know->F.Att	0.182	0.031	5.823	0.000
H5	F.Att->M.SN	0.297	0.033	9.126	0.000
H6	F.Att->F.Supp	0.840	0.018	46.282	0.000
H7	F.Supp->M.Att	0.886	0.013	69.099	0.000
H8	M.Att->M.Int	0.142	0.047	3.035	0.003
H9	M.SN->M.Int	0.097	0.049	1.980	0.048
H10	M.SE->M.Int	0.692	0.105	6.574	0.000
H11	M.PC->M.Int	0.719	0.095	7.572	0.000
H12	M.SE->EBF	1.123	0.092	12.176	0.000
H13	M.PC->EBF	1.253	0.078	15.965	0.000
H14	M.Int->EBF	0.243	0.036	6.804	0.000
Control Variables	Profession->EBF	−0.082	0.038	2.165	0.031
	Residence->EBF	−0.044	0.028	1.574	0.116
	Education->EBF	−0.005	0.031	0.178	0.859
	Income->EBF	0.088	0.040	2.198	0.028

Note: SD = Standard Deviation; Please see Table 2 for variable names elaboration.

## Data Availability

Data can be made available on request to the principal Investigator.

## Appendix A

**Table A1:** Path coefficients of SEM (using cesarean delivery sub-sample).

Hypotheses	Path	β	SD	*t*-Value	*p*-Value
H1	M.Know->M. Att	0.490	0.047	1.277	0.020
H2	M.Exp->M.Att	0.001	0.045	0.015	0.988
H3	F.Know->M.Know	0.863	0.025	34.400	0.000
H4	F.Know->F.Att	0.081	0.089	0.905	0.366
H5	F.Att->M.SN	0.280	0.072	3.866	0.000
H6	F.Att->F.Supp	0.717	0.061	11.836	0.000
H7	F.Supp->M.Att	0.881	0.032	27.258	0.000
H8	M.Att->M.Int	0.196	0.105	1.861	0.036
H9	M.SN->M.Int	0.026	0.102	0.252	0.801
H10	M.SE->M.Int	0.200	0.124	1.607	0.109
H11	M.PC->M.Int	0.551	0.071	7.703	0.000
H12	M.SE->EBF	0.231	0.148	1.556	0.120
H13	M.PC->EBF	0.877	0.077	11.350	0.000
H14	M.Int->EBF	0.033	0.060	0.552	0.034
Control Variables	Profession->EBF	−0.071	0.075	0.951	0.342
	Residence->EBF	−0.043	0.032	1.318	0.188
	Education->EBF	0.032	0.045	0.702	0.483
	Income->EBF	0.008	0.049	0.166	0.869

Note: SD = Standard Deviation.

**Table A2:** Path coefficients of SEM (using vaginal delivery sub-sample).

Hypotheses	Path	β	SD	*t*-Value	*p*-Value
H1	M.Know->M. Att	0.024	0.031	0.790	0.043
H2	M.Exp->M.Att	0.010	0.032	0.312	0.755
H3	F.Know->M.Know	0.845	0.023	36.175	0.000
H4	F.Know->F.Att	0.181	0.076	2.381	0.018
H5	F.Att->M.SN	0.285	0.074	3.873	0.000
H6	F.Att->F.Supp	0.861	0.024	36.204	0.000
H7	F.Supp->M.Att	0.910	0.015	61.354	0.000
H8	M.Att->M.Int	0.524	0.068	7.664	0.000
H9	M.SN->M.Int	0.124	0.079	1.564	0.118
H10	M.SE->M.Int	0.078	0.072	1.083	0.279
H11	M.PC->M.Int	0.396	0.071	5.553	0.000
H12	M.SE->EBF	0.030	0.015	1.981	0.048
H13	M.PC->EBF	0.984	0.008	129.326	0.000
H14	M.Int->EBF	0.030	0.016	1.904	0.018
Control Variables	Profession->EBF	−0.031	0.019	1.628	0.104
	Residence->EBF	−0.012	0.012	0.982	0.326
	Education->EBF	−0.012	0.015	0.776	0.438
	Income->EBF	0.043	0.018	2.376	0.018

Note: SD = Standard Deviation.

**Table A3:** Parental demographic characteristics in respect to total participants.

		Mothers’ Demographic Characteristics	Percentage (of Total)	Fathers’ Demographic Characteristics	Percentage (of Total)
Age	18–25 Years	12	3.6	20	6.0
	26–33 Years	172	51.8	126	38.0
	34–41 Years	144	43.4	168	50.6
	42–49 Years	4	1.2	18	5.4
Number of Alive Children	1 Children	232	69.9	232	69.9
	2 Children’s	79	23.8	79	23.8
	3 Children’s	e	5.7	19	5.7
	4 Children’s	2	0.6	2	0.6
Resident of the Division	Sylhet	8	2.4	8	2.4
	Mymensingh	6	1.8	6	1.8
	Khulna	34	10.2	34	10.2
	Rangpur	10	3.0	10	3.0
	Chittagong	45	13.6	45	13.6
	Barisal	7	2.1	7	2.1
	Rajshahi	19	5.7	19	5.7
	Dhaka	203	61.1	203	61.1
Religion	Christians	12	3.6	12	3.6
	Buddhism	4	1.2	4	1.2
	Hindu	43	13.0	43	13.0
	Islam	273	82.2	273	82.2
Profession	Searching for work/unemployed	33	9.9	8	2.4
	Housewife	76	22.9		
	Business			78	23.5
	Employed for wage			69	20.8
	Public Sector Employee	116	34.9	79	23.8
	Corporate Employee	107	32.2	98	29.5
Education	Less than high school degree (Less than SSC)	20	6.0	34	10.2
	High school degree (HSC)	66	19.9	11	3.3
	Bachelor degree	74	22.3	97	29.2
	Masters’ degree and above	172	51.8	190	57.2
Income Per Month	<$100	115	34.6	47	14.2
	$100–$300	38	11.4	58	17.5
	$300–$500	106	31.9	101	30.4
	$500–$800	56	16.9	105	31.6
	>$800	17	5.1	21	6.3

Source: Authors’ calculations.

**Table A4:** Variance influential factor (VIF) of the variables.

Variables	F.Know	F.Att	F.Supp	M.Know	M.Exp	EBF	M.Int	M.Att	M.PC	M.SN	M.SE
F.Know		1.000		1.000							
F.Att			1.000					1.191		1.089	
F.Supp								1.169			
M.Know								1.762			
M.Exp								1.017			
EBF											
M.Int						1.230					
M.Att							1.108				
M.PC						1.209	1.195	1.272		1.083	
M.SN							1.586	1.838			
M.SE						1.026	1.431			1.030	
